# Recent Development of Heat and Mass Transport in the Presence of Hall, Ion Slip and Thermo Diffusion in Radiative Second Grade Material: Application of Micromachines

**DOI:** 10.3390/mi13101566

**Published:** 2022-09-21

**Authors:** V. V. L. Deepthi, Maha M. A. Lashin, N. Ravi Kumar, Kodi Raghunath, Farhan Ali, Mowffaq Oreijah, Kamel Guedri, El Sayed Mohamed Tag-ElDin, M. Ijaz Khan, Ahmed M. Galal

**Affiliations:** 1 Department of Mathematics, CVR College of Engineering, Hyderabad 500039, India; 2 College of Engineering, Princess Nourah Bint Abdulrahman University, Riyadh 84428, Saudi Arabia; 3 Department of Mathematics, Malla Reddy Engineering College (Autonomous), Medchal 500100, India; 4 Department of Humanities and Sciences, Bheema Institute of Technology and Science, Adoni 518301, India; 5 Department of Mathematical Sciences, Federal Urdu University of Arts, Sciences & Technology, Gulshan-e-Iqbal Karachi 75300, Pakistan; 6 Mechanical Engineering Department, College of Engineering and Islamic Architecture, Umm Al-Qura University, Makkah 21955, Saudi Arabia; 7 Research Unity: Materials, Energy and Renewable Energies, Faculty of Science of Gafsa, University of Gafsa, Gafsa 2100, Tunisia; 8 Faculty of Engineering and Technology, Future University in Egypt, New Cairo 11835, Egypt; 9 Department of Mechanical Engineering, American Lebanese University, Beirut 1102, Lebanon; 10 Mechanical Engineering Department, College of Engineering, Prince Sattam Bin Abdulaziz University, Wadi Addawaser 11991, Saudi Arabia; 11 Production Engineering and Mechanical Design Department, Faculty of Engineering, Mansoura University, Mansoura 35516, Egypt

**Keywords:** diffusion thermo effect, radiation absorption, porous media, Hall and ion slip effects

## Abstract

This article describes the incompressible two-dimensional heat and mass transfer of an electrically conducting second-grade fluid flow in a porous medium with Hall and ion slip effects, diffusion thermal effects, and radiation absorption effects. It is assumed that the fluid is a gray, absorbing–emitting but non-scattering medium and the Rosseland approximation is used to describe the radiative heat flux in the energy equation. It is assumed that the liquid is opaque and absorbs and emits radiation in a manner that does not result in scattering. It is considered an unsteady laminar MHD convective rotating flow of heat-producing or absorbing second-grade fluid across a semi-infinite vertical moving permeable surface. The profiles of velocity components, temperature distribution, and concentration are studied to apply the regular perturbation technique. These profiles are shown as graphs for various fluid and geometric parameters such as Hall and ion slip parameters, radiation absorption, diffusion thermo, Prandtl number, Schmidt number, and chemical reaction rate. On the other hand, the skin friction coefficient and the Nusselt number are determined by numerical evaluation and provided in tables. These tables are then analysed and debated for various values of the flow parameters that regulate it. It may be deduced that an increase in the parameters of radiation absorption, Hall, and ion slip over the fluid region increases the velocity produced. The resulting momentum continually grows to a very high level, with contributions from the thermal and solutal buoyancy forces. The temperature distribution may be more concentrated by raising both the heat source parameter and the quantity of radiation. When one of the parameters for the chemical reaction is increased, the whole fluid area will experience a fall in concentration. Skin friction may be decreased by manipulating the rotation parameter, but the Hall effect and ion slip effect can worsen it. When the parameter for the chemical reaction increases, there is a concomitant rise in the mass transfer rate.

## 1. Introduction

A few decades ago, the majority of academics focused more of their attention on MHD fluid flow difficulties. Still, they overlooked the Hall current and the Ion-slip current in Ohm’s law. The flow of MHD heat and mass transfer in the presence of a high magnetic field is essential in solving a wide variety of engineering issues, including those involving astronomy, chemical engineering, and geophysics. There is a good chance that Hall and ion-slip currents play an essential role in the flow of laboratory plasma. As a result, a recent suggestion has been made to investigate the heat and mass transfer of a free-convection MHD flow that goes through an infinite vertical porous plate while considering Hall and ion slip currents. Recent research on magnetohydrodynamic (MHD)-driven flows, on the other hand, is focused on the firm application of a magnetic field since this causes many other complicated phenomena, such as the Hall current, ion slip, Joule heating, and so on (Sutton and Sherman [1]). This application’s relevance may be observed in many different engineering processes, such as magnetic fusion systems, energy generators, Hall sensors, and Hall accelerators, as well as in specific astrophysical applications. Abo-Eldahab et al. [2] investigated the combined effects of the hall and ion-slip currents on free convective flow across a semi-infinite vertical plate. Ref. [2] Srinivasacharya and Kaladhar [3] used the homotopy analysis method to investigate the effect of Hall and Ion-slip currents on fully developed electrically conducting fluid flow between vertical parallel plates in the presence of a temperature-dependent heat source. This study was conducted in the presence of a temperature-dependent heat source. After that, Darbhasayanam et al. [4] looked at how Hall and ion slip currents affect the flow of electrically conducting couple stress fluid between two circular cylinders when a temperature-dependent heat source is present. Tani [5] researched the steady motion of an electrically conducting viscous liquid in the presence of Hall current. Motsa and Shateyi [6] performed a numerical analysis on magnetomicropolar fluid flow, heat and mass transfer with suction through porous material to determine the impacts of Hall currents, ion-slip currents, and changing thermal diffusivity on these processes. Attia [7] used an analytical approach to research the flow of a dusty fluid when Hall and ion slip current were present. The flow of a magnetohydrodynamic boundary layer was examined by Ghosh [8] across a stretched sheet while a chemical reaction was taking place. The chemically reactive second grade via porous saturated space was investigated by Raghunath et al. [9] using a perturbation technique. Raghunath et al. [10] have investigated the effects of Soret, Rotation, Hall, and Ion Slip on the unsteady flow of a Jeffrey fluid through a porous medium. At the same time, heat is absorbed, and chemical reactions occur. Sibanda and Makinde [11] have investigated the hydromagnetic steady flow and heat transfer characteristics of an incompressible viscous electrically conducting fluid past a rotating disk in a porous medium with ohmic heating, Hall current and viscous dissipation.

The study of magnetohydrodynamics in the presence of heat and mass transfer, as well as radiation and diffusion, has captured the interest of a sizable number of academics as a result of the wide variety of applications it may be put to use. In the fields of astrophysics and geophysics, it is used to examine the structures of stars and the sun, radio transmission via the ionosphere, and other such phenomena. Its uses may be found in engineering, such as in MHD pumps, MHD bearings, and other places. The process of mass transfer is also quite prevalent in theoretical discussions on the structure of stars, and its effects may be noticeable on the sun’s surface. The investigation of the impact of a magnetic field plays a significant role in free convection flow, particularly in the case of liquid metals, electrolytes, and ionized gases. The thermal physics of hydromagnetic issues with mass transport has vast implications in the field of power engineering. Various industrial and environmental activities involve the presence of radiative fluxes. A few examples of this are heating and cooling chambers, energy processes involving the burning of fossil fuels, evaporation from huge open water reservoirs, astrophysical flows, solar power technologies, and the re-entry of space vehicles. Seth and Sarkar [12] investigated the influence of an induced magnetic field on the hydromagnetic natural convection flow of a chemically reacting fluid across a moving vertical plate with ramping wall temperature. The effects of an induced magnetic field on the flow of a free convective channel were studied by Sarveshanand and Singh [13]. Sarma and Pandit [14] investigated the effects of thermal radiation, chemical reactions, and generated magnetic fields on MHD mixed convection flow across a vertical porous plate. Following that, Ojjela et al. [15] investigated the effects that thermophoresis and an induced magnetic field had on a mixed convective Jeffrey fluid contained between two porous plates. Jha and Aina [16] show the interplay of conducting and non-conducting walls on the MHD natural convection flow in a vertical micro-channel with an induced magnetic field. Shaw et al. [17] have studied impact of Entropy Generation and Nonlinear Thermal Radiation on Darcy–Forchheimer Flow of MnFe_2_O_4_-Casson/Water Nanofluid due to a Rotating Disk. Very recently Sharma [18] has studied FHD flow and heat transfer over a porous rotating disk accounting for Coriolis force along with viscous dissipation and thermal radiation. Ram et al. [19] have possessed a Ferrofluid flow over a moving plate in a porous medium is theoretically investigated by solving the boundary layer equations with boundary conditions using Neuringer–Rosensweig model. Mahantesh et al. [20] have studied impacts of a novel exponential space dependent heat source on MHD slip flow of carbon nanoliquids past a stretchable rotating disk. The flow is created due to rotation and stretching of the disk. Vijay and Sharma [21] have studied heat and mass transfer of ferrofluid flow between co-rotating stretchable disks with geothermal viscosity.

The investigation of first-order chemical reactions that include simultaneous heat and mass transfer has attracted many researchers in recent years. It has been the focus of considerable stress in recent times. Evaporation at the surface of a water body and heat and mass transfer all take place concurrently in several different processes, including energy transfer in a wet cooling tower, flow in a desert cooler, and energy transfer in a desert cooler. Some of the uses of this flow may be found in many different sectors, such as the power industry. One of the techniques of producing electrical energy is to directly extract it from moving conducting fluid, which is one of the applications of this flow. Studying heat production or absorption in flowing fluids is vital in issues involving chemical processes that dissociate fluids. These problems may be broken down into two categories: The effects that the creation of heat might cause could potentially change the temperature distribution and, as a result, the pace at which particles are deposited in nuclear reactors, electronic chips, and semiconductors wafers. It is interesting to study the effects of a magnetic field on the temperature distribution and heat transfer when the fluid is not only an electrical conductor but also capable of emitting and absorbing radiation because some fluids are also capable of emitting and absorbing thermal radiation. Because of this, heat transmission through thermal radiation is becoming more significant as we become more concerned with space applications and higher operating temperatures. Recent research conducted by Raghunath and colleagues [22,23,24] investigated the impact of chemical reactions on different flow geometries. The effects of Soret on the unsteady free convection flow of a viscous incompressible fluid through a porous medium with high porosity bounded by a vertical infinite moving plate have been discussed by Ramachandra et al. [25]. This flow occurs under thermal diffusion, a chemical reaction, and a heat source. Raghunath and Mohanaramana [26] have researched Hall, Soret, and rotational effects on unsteady MHD rotating flow of a second-grade fluid through a porous media in the presence of chemical reaction and aligned magnetic field. Their findings were published not too long ago.

In the fields of geophysics, petrochemical engineering, meteorology, oceanography, and aeronautics, the notion of fluid flow, heat and mass transfer inside a rotating environment plays a very significant part in the applications of these sciences. Applications in geophysics and fluid engineering are where the impetus for scientific study on rotating fluid systems first began. This is where the field has been propelled forward. The effect of rotation significantly impacts the motion of the atmospheres of both planets and the earth. This has implications for several different elements of atmosphere motion. The theory of rotational flow is used for figuring out the fluid’s viscosity, constructing centrifugal devices like the turbine, and other similar activities. Ali et al. [27] have studied Saleel, Entropy Generation Analysis of Peristaltic Flow of Nanomaterial in a Rotating Medium through Generalized Complaint Walls of Micro-Channel with Radiation and Heat Flux Effects. Ali et al. [28] have analyzed the slippage phenomenon in hydromagnetic peristaltic rheology with Hall current and viscous dissipation. Awais et al. [29] possessed Convective and peristaltic viscous fluid flow with variable viscosity, J. Engin. Thermophys. Ali et al. [30] investigated Oscillatory Flow in a Porous Channel with Porous Medium and Small Suction. Ali et al. [31] have studied Oscillatory flow of second grade fluid in cylindrical tube. Ilya et al. [32] have possessed Heat source and sink effects on periodic mixed convection flow along the electrically conducting cone inserted in porous medium.

Raghunath et al. [10] conducted research not too long ago in which they studied the effect of Hall and ion-slip currents on the unsteady MHD flows of a viscous, incompressible, and electrically conducting fluid that was occurring between two vertical plates in a rotating system when the lower plate was impulsively started. The work done by Raghunath et al. [10] for the diffusion thermal and Second grade fluid cases will be extended even further with the help of this effort. After obtaining the flow equations in a dimensionless form using the perturbation technique, the flow equations are then analytically solved under the appropriate conditions. The graphical representation and subsequent discussion of the influence of various flow parameters on the fluid velocity, volume flow rate, and surface friction are shown below. The significance of the findings acquired from this research is that they provide the criteria for verifying the accuracy of various numerical or empirical methodologies. In addition, the results that were produced from this study have the potential to be used in the fields of fluid mechanics and heat transport.

## 2. Formulation of the Problem

**Figure 1:**
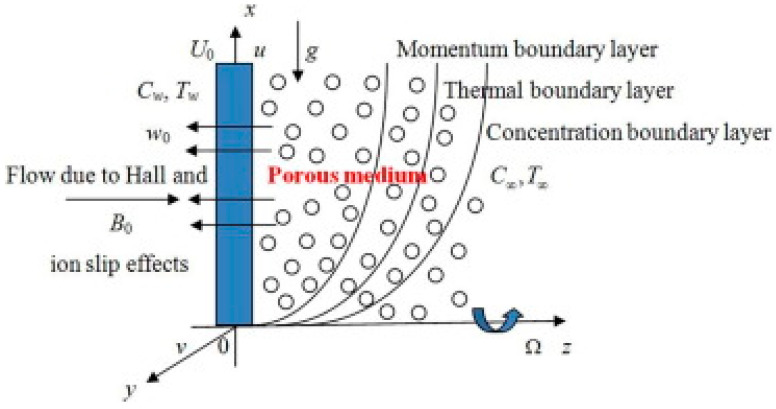
Flow diagram.

We considered the heat and mass transfer of an unsteady two-dimensional MHD convective flow of a viscous laminar heat initiating second-grade liquid over a semi-infinite longitudinal moveable porous layer engrained in consistent permeable material. We adapted to a homogeneous transverse magnetic field. We do this while considering Hall and ion slip consequences. The Cartesian coordinate system is selected so that the *x*-axis is maintained along the wall in the direction of upward movement, and the *z*-axis is perpendicular to this orientation. A magnetic field with strength of B_0_ and a uniform intensity is moving in a direction that is perpendicular to the flow. In their original, undisturbed states, the fluid and the plate are both rotating in a fixed orientation relative to the perpendicular to the plate at a constant angular velocity. At the surface, the temperature and concentration are subject to random fluctuations; this is true for both the fluid and the plate. The investigational challenge may be seen by looking at the physical model shown in Figure 1.

The fluid properties are assumed to be constant except that the influence of density variation with temperature has been considered only in the body-force term. The concentration of diffusing species is very small in comparison to other chemical species, the concentration of species far from the wall, $C_{\alpha }$ s infinitesimally small and hence the Soret and Dufour effects are neglected. The chemical reactions are taking place in the flow and all thermophysical properties are assumed to be constant of the linear momentum equation which is approximated according to the Boussinesq approximation. Due to the semiinfinite plane surface assumption, the flow variables are functions of z and the time t only. The flow governing equations and boundary conditions as followed by Raghunath et al. [10].

(1) $$\frac{\partial u}{\partial x}+\frac{\partial u}{\partial y}=0$$

(2) $$\begin{array}{l} \frac{\partial u}{\partial t}+w\frac{\partial u}{\partial z}-2\;\Omega \;v=-\frac{1}{\rho }\frac{\partial p}{\partial x}+v\frac{\partial^{2}u}{\partial z^{2}}+\frac{\alpha }{\rho }\frac{\partial^{3}u}{\partial z^{2}\partial t}+\frac{B_{0}\;J_{y}}{\rho }-\frac{v\;}{k}u+g\beta (T-T_{\infty \;}^{{}^{**}})\;+g\beta^{*}(C-C_{\infty }) \end{array}$$

(3) $$\frac{\partial v}{\partial t}+w\frac{\partial v}{\partial z}+2\;\Omega \;u=-\frac{1}{\rho }\frac{\partial p}{\partial y}+v\frac{\partial^{2}v}{\partial z^{2}}+\frac{\alpha }{\rho }\frac{\partial^{3}v}{\partial z^{2}\partial t}-\frac{B_{0}\;J_{x}}{\rho }-\frac{v\;}{k}v$$

(4) $$\frac{\partial T}{\partial t}+w\frac{\partial T}{\partial z}=\frac{k_{1}}{\rho C_{p}}\frac{\partial^{2}T}{\partial z^{2}}-\frac{1}{\rho C_{p}}\frac{\partial q_{r}}{\partial z}-\frac{Q_{0}}{\rho C_{p}}(T-T_{\infty })+Q_{1}(C-C_{\infty })+\frac{DK_{T}}{C_{s}C_{p}}\frac{\partial^{2}C}{\partial z^{2}}$$

(5) $$\frac{\partial C}{\partial t}+w\frac{\partial C}{\partial z}=D\frac{\partial^{2}C}{\partial z^{2}}-K_{c}(C-C_{\infty })$$

The proper boundary requirements for the velocity, temperature, and concentration distributions are provided if the assumptions outlined above hold.

(6) $$u=U_{0},\;\;\;\;\;v=0,\;\;T=T_{w}+\varepsilon \;(T_{w}-T_{\infty })e^{iwt},\;\;C=C_{w}+\varepsilon (C_{w}-C_{\infty })e^{iw\;t}\;\;\;\mathrm{at}\;\;z=0$$

(7) $$u\to U_{\infty },\;\;\;\;\;v\to 0,\;\;\;\;\;T\to \;\;T_{\infty }\;\;\;\;\;\;\;\;\;\;C\to C_{\infty }\;\;\;\;\;\;\;\;\;\mathrm{as}\;\;\;\;\;\;\;z\to \infty$$

In light of the fact that the solution to the continuity equation is either a constant or a function of time, we will assume that.

w = −w^1^_0_(1 + Aεe^iwt^) (8)

where A is a real positive constant, ε and Aε are small, less than unity, w_0_ is the scale of the suction velocity which has a non-zero positive constant.

The Rosseland approximation can be used for the radiative heat flux vector *q_r_* because, for an optically thick fluid, in addition to emission, there is also self-absorption. Since the absorption coefficient is typically wavelength dependent and significant, we can use the Rosseland approximation. Therefore, *q_r_* may be deduced from

(9) $$q_{r}=\frac{-4\sigma_{1}}{3k_{1}}\frac{\partial^{2}T^{4}}{\partial z}$$

where *k*_1_ is the Rosseland mean absorption co-efficient and *σ*_1_ is the Stefan–Boltzmann constant.

We assume that the temperature differences within the flow are sufficiently small so that *T*^4^ can be expressed as a linear function. Using Taylor’s series, we expand *T*^4^ to the free stream temperature *T* and neglect higher order terms. This results in the following approximation:

(10) $$T^{4}\approx 4T_{\infty }^{3}T-3T_{\infty }^{4}$$

As a result, we have

(11) $$\frac{\partial q_{r}}{\partial z}=\frac{-16\sigma_{1}T_{\infty }^{3}}{3k_{1}}\frac{\partial^{2}T}{\partial z^{2}}$$

Equation (4), derived from Equations (10) and (11), may be simplified to

(12) $$\frac{\partial T}{\partial t}+w\frac{\partial T}{\partial z}=\frac{k_{1}}{\rho C_{p}}\frac{\partial^{2}T}{\partial z^{2}}-\frac{Q_{0}}{\rho C_{p}}(T-T_{\infty })+\frac{1}{\rho C_{p}}\frac{16\sigma_{1}T_{\infty }^{3}}{3k_{1}}\frac{\partial^{2}T}{\partial z^{2}}+Q_{1}(C-C_{\infty })+\frac{DK_{T}}{C_{s}C_{p}}\frac{\partial^{2}C}{\partial z^{2}}$$

Because it is presumed that the frequency of electron–atom collisions is very high, it is impossible to ignore Hall and ion slip currents. Therefore, the velocity in the y-direction is caused by Hall currents and ion slip currents. When the magnitude of the magnetic field is exceptionally great, the generalised law of Ohm is adjusted such that it takes into account the Hall effect as well as the ion slip effect (Sutton and Sherman [1]),

(13) $$J=\sigma \left(E+V\times B\right)-\frac{\omega_{e}\;\tau_{e}}{B_{0}}\left(J\times B\right)+\frac{\omega_{e}\;\tau_{e}\;\beta_{i}}{B_{0}^{2}}\left(\left(J\times B\right)\times B\right)$$

Further it is assumed that $\beta_{e}=\omega_{e}\tau_{e}\sim O\left(1\right)$ and $\beta_{i}=\omega_{i}\tau_{i}\ll 1,$ In the Equation (13) the electron pressure gradient, the ion-slip and thermo-electric effects are neglected. We also assume that the electric field *E = 0* under assumptions reduces to

(14) $$\left(1+\beta_{i}\beta_{e}\right)\;J_{x}+\beta_{e}J_{y}=\sigma B_{0}v$$

(15) $$\left(1+\beta_{i}\beta_{e}\right)\;J_{y}+\beta_{e}J_{x}=-\sigma B_{0}v$$

On solving above Equations (14) and (15), we get

(16) $$J_{x}=\sigma B_{0}\left(\alpha_{2}u+\alpha_{1}v\right)$$

(17) $$J_{y}=-\sigma B_{0}\left(\alpha_{2}v-\alpha_{1}u\right)$$

$$Where\;\alpha_{1}=\frac{1+\beta_{e}\;\beta_{i}}{{\left(1+\beta_{e}\;\beta_{i}\right)}^{2}+\beta_{e}^{2}},\;\alpha_{2}=\frac{\beta_{e}\;}{{\left(1+\beta_{e}\;\beta_{i}\right)}^{2}+\beta_{e}^{2}}$$

when Equations (16) and (17) are introduced into (2) and (3), respectively, the equations that are produced are,

(18) $$\begin{array}{l} \frac{\partial u}{\partial t}+w\frac{\partial u}{\partial z}-2\;\Omega \;v=-\frac{1}{\rho }\frac{\partial p}{\partial x}+v\frac{\partial^{2}u}{\partial z^{2}}+\frac{\alpha }{\rho }\frac{\partial^{3}u}{\partial z^{2}\partial t}+\frac{\sigma B_{0}^{2}\left(\alpha_{2}v-\alpha_{1}u\right)}{\rho }-\frac{v\;}{k}u+g\beta (T-T_{\infty \;}^{{}^{**}})\;+g\beta^{*}(C-C_{\infty }) \end{array}$$

(19) $$\frac{\partial v}{\partial t}+w\frac{\partial v}{\partial z}+2\;\Omega \;u=-\frac{1}{\rho }\frac{\partial p}{\partial y}+v\frac{\partial^{2}v}{\partial z^{2}}+\frac{\alpha }{\rho }\frac{\partial^{3}v}{\partial z^{2}\partial t}-\frac{\sigma B_{0}^{2}\left(\alpha_{2}u+\alpha_{1}v\right)}{\rho }-\frac{v\;}{k}v$$

Fusing Equations (2) and (3), let q=u+iv   and   ξ=x−iy, we obtain

(20) $$\begin{array}{l} \frac{\partial q}{\partial t}+w\frac{\partial q}{\partial z}+2i\;\Omega \;q=-\frac{1}{\rho }\frac{\partial p}{\partial \xi }+v\frac{\partial^{2}q}{\partial z^{2}}+\frac{\alpha }{\rho }\frac{\partial^{3}q}{\partial z^{2}\partial t}+\frac{\sigma B_{0}^{2}\left(\alpha_{2}v-\alpha_{1}u\right)}{\rho }-\frac{v\;}{k}q+g\beta (T-T_{\infty \;}^{{}^{**}})\;+g\beta^{*}(C-C_{\infty }) \end{array}$$

Beyond the border layer, Equation (20) provides

(21) $$-\frac{1}{\rho }\frac{\partial p}{\partial \xi }=\frac{dU_{\infty }}{\partial t}+\;\frac{v}{k}U_{\infty }+\frac{\sigma B_{0}^{2}}{\rho }U_{\infty }$$

To standardise the mathematical representation of the physical issue, we will introduce the non-dimensional quantities and parameters listed below.

(22) $$\begin{array}{l} q*=\frac{q}{w_{0}},w*=\frac{w}{w_{0}},\;\;z^{*}=\frac{w_{0}z}{v},\;\;U_{0}^{*}=\frac{U_{0}}{w_{0}},\;U_{\infty }^{*}=\frac{U_{\infty }}{w_{0}},\;\;t^{*}=\frac{t\;w_{0}^{2}}{v},\theta =\frac{T-T_{\infty }}{T_{w}-T_{\infty }},\Phi =\frac{C-C_{\infty }}{C_{w}-C_{\infty }},\\ M^{2}=\frac{\sigma B_{0}^{2}v}{\rho w_{0}^{2}},\mathrm{Pr}=\frac{v\;\rho C_{p}}{k_{1}}=\frac{v}{\alpha },r=\frac{vg\beta (T_{w}-T_{\infty })}{w_{0}^{3}},Gm=\frac{vg\beta^{*}(C_{w}-C_{\infty })}{w_{0}^{3}},\\ K=\frac{w_{0}^{2}k}{v^{2}},\;Sc=\frac{v}{D},R=\frac{\Omega v}{w_{0}^{2}},H=\frac{vQ_{0}}{\rho C_{p}w_{0}^{2}},\;S=\frac{w_{0}^{2}\;\alpha_{1}}{\rho \;v^{2}},\;\;K_{c}=\frac{\;K_{c}v}{w_{0}^{2}},F=\frac{16\sigma^{*}T_{\infty }^{{}^{3}}}{3kk_{1}}\\ Du=\frac{DK_{T}}{\nu C_{s}C_{p}}\frac{\left(C_{w}-C_{\infty }\right)}{\left(T_{w}-T_{\infty }\right)},\;Q_{1}=\frac{v\;Q_{1}(C_{w}-C_{\infty })}{(T_{w}-T_{\infty })\;w_{0}^{2}} \end{array}$$

By exploiting variables that are not dimensional, the three governing Equations (5), (12), and (20), reduced to

(23) $$\frac{\partial q}{\partial t}-(1+A\varepsilon \;e^{iwt})\frac{\partial q}{\partial z}=\frac{dU_{\infty }}{dt}+\frac{\partial^{2}q}{\partial z^{2}}+S\frac{\partial^{3}q}{\partial z^{2}\partial t}-\lambda \;q+Gr\;\theta \;+Gm\;\;\Phi$$

$$Where\;\lambda \;\;=M^{2}(\alpha_{1}+i\alpha_{2})+2iR+\frac{1}{K}$$

(24) $$\frac{\partial \theta }{\partial t}-(1+A\varepsilon e^{iwt})\frac{\partial \theta }{\partial z}=-\frac{\left(1+F\right)}{\mathrm{Pr}}\frac{\partial^{2}\theta }{\partial z^{2}}-H\;\theta +Q_{1}\Phi +D_{u}\frac{\partial^{2}\Phi }{\partial z^{2}}$$

(25) $$\frac{\partial \Phi }{\partial t}-(1+A\varepsilon e^{iwt})\frac{\partial \Phi }{\partial z}=\frac{1}{Sc}\frac{\partial^{2}\Phi }{\partial z^{2}}-K_{c}\Phi \;$$

The equations that relate to the boundary conditions are as follows:

(26) $$q=U_{0},\;\;\;\;\theta =1+\varepsilon \;e^{iwt},\;\;\Phi =1+\varepsilon e^{iw\;t}\;\;\;\;\;\;\;\;\;\;\;\;\;\;\;\;\;\;\;\;\mathrm{at}\;\;z=0$$

(27) q=0,    θ=0,    Φ=0                                    as       z→∞

## 3. Solution of the Problem

The expressions (23) and (25) portray a set of partial differential equations that cannot be solved in closed form; nonetheless, if the equations can be reduced to a set of ordinary differential equations in dimensionless form, then the equations can be solved analytically. This can be accomplished by expressing the velocity, temperature, and concentration as,

(28) $$q=q_{0}\left(z\right)+\varepsilon e^{nt}q_{1}\left(z\right)+O\left(\varepsilon^{2}\right)$$

(29) $$\theta =\theta_{0}\left(z\right)+\varepsilon e^{nt}\theta_{1}\left(z\right)+O\left(\varepsilon^{2}\right)$$

(30) $$\phi =\phi_{0}\left(z\right)+\varepsilon e^{nt}\phi_{1}\left(z\right)+O\left(\varepsilon^{2}\right)$$

The following pairs of equations are obtained by substituting the Equations (28)–(30) into the Equations (23)–(25) by equating the harmonic and non-harmonic components, as well as the neglecting and higher order terms of, and by obtaining the following:

(31) $$\frac{\partial^{2}q_{0}}{\partial z^{2}}+\frac{\partial q_{0}}{\partial z}-\lambda \;q_{0}=-Gr\;\theta_{0}\;-Gm\;\;\Phi_{0}$$

(32) $$\left(1+Siw\right)\frac{\partial^{2}q_{1}}{\partial z^{2}}+\frac{\partial q_{1}}{\partial z}-\left(\lambda +iw\right)\;q_{1}=-Gr\;\theta_{1}\;-Gm\;\;\Phi_{1}-A\frac{\partial q_{0}}{\partial z}$$

(33) $$\frac{\partial^{2}\theta_{0}}{\partial z^{2}}+\mathrm{Pr}\frac{\partial \theta_{0}}{\partial z}-\left(H\;+F\right)\mathrm{Pr}\;\theta_{0}=-\mathrm{Pr}\;\left(Q_{1}\Phi_{0}+D_{u}\;\frac{\partial^{2}\Phi_{0}}{\partial z^{2}}\right)$$

(34) $$\frac{\partial^{2}\theta_{1}}{\partial z^{2}}+\mathrm{Pr}\frac{\partial \theta_{1}}{\partial z}-\left(iw+H+F\right)\;\mathrm{Pr}\;\theta_{1}=-\mathrm{Pr}\;\left(A\frac{\partial \theta_{0}}{\partial z}+Q_{1}\Phi_{1}+D_{u}\frac{\partial^{2}\Phi_{1}}{\partial z^{2}}\right)$$

(35) $$\frac{\partial^{2}\phi_{0}}{\partial z^{2}}+Sc\frac{\partial \phi }{\partial z}-Sc\;Kc\;\phi_{0}=0$$

(36) $$\frac{\partial^{2}\phi_{1}}{\partial z^{2}}+Sc\frac{\partial \phi_{1}}{\partial z}-\left(iw+K_{c}\right)Sc\;\phi_{1}=-A\;Sc\frac{\partial \phi_{0}}{\partial z}$$

The requirements that relate to each border are as follows:

(37) $$q_{0}=U_{0},q_{1}=0,\;\;\;\;\theta_{0}=1,\;\;\;\theta_{1}=1,\;\;\phi_{0}=1\;,\;\;\phi_{1}=1\;\;\;\;\;\;\;\;\;\;\;\;\;\;\;\;\;\mathrm{at}\;\;z=0$$

(38) $$q_{0}=0,q_{1}=0,\;\;\;\;\theta_{0}=0,\;\;\;\theta_{1}=0,\;\;\phi_{0}=0,\;\;\phi_{1}=0\;\;\;\;\;\;\;\;\;\;\;\;\;\;\;\;\;\;\mathrm{as}\;\;\;\;\;\;\;z\to \infty$$

By applying the initial conditions (37) and (38) and then solving Equations (31)–(36), one obtains the following approach:

(39) $$\varphi_{0}=\exp(-m_{1}z)$$

(40) $$\varphi_{1}=b_{1}\exp(-m_{1}z)+b_{2}\exp(-m_{2}z)\;$$

(41) $$\theta_{0}=b_{3}\exp(-m_{1}z)+b_{4}\exp(-m_{3}z)$$

(42) $$\theta_{1}=b_{5}\exp(-m_{1}z)+b_{6}\exp(-m_{2}z)+b_{7}\exp(-m_{3}z)+b_{8}\exp(-m_{4}z)$$

(43) $$q_{0}=b_{9}\exp(-m_{1}z)+b_{10}\exp(-m_{3}z)+b_{11}\exp(-m_{5}z)$$

(44) $$\begin{array}{l} q_{1}=b_{12}\exp(-m_{1}z)+b_{13}\exp(-m_{2}z)+b_{14}\exp(-m_{3}z)+b_{15}\exp(-m_{4}z)+\\ \;\;\;\;\;\;\;\;\;\;\;\;\;\;\;\;\;\;\;\;\;\;\;\;\;\;\;\;\;\;\;\;\;\;\;\;\;\;\;\;b_{16}\exp(-m_{5}z)+b_{17}\exp(-m_{6}z) \end{array}$$

Substituting Equations (39)–(44) into Equations (28)–(30), we acquire the velocity temperature and concentration

(45) $$\begin{array}{l} q=b_{9}\exp(-m_{1}z)+b_{10}\exp(-m_{3}z)+b_{11}\exp(-m_{5}z)+\\ \;\;\;\;\;\;\;\;\;\;\;\;\;\;\;\;\;\;\;\;\;\;\;\;\;\;\;\;\;\;\;\;\;\;\varepsilon e^{iwt}\left(\begin{array}{l} b_{12}\exp(-m_{1}z)+b_{13}\exp(-m_{2}z)+b_{14}\exp(-m_{3}z)+\\ b_{15}\exp(-m_{4}z)+\;\;b_{16}\exp(-m_{5}z)+b_{17}\exp(-m_{6}z) \end{array}\right) \end{array}$$

(46) $$\begin{array}{l} \theta =b_{3}\exp(-m_{1}z)+b_{4}\exp(-m_{3}z)\;+\varepsilon e^{iwt}(b_{5}\exp(-m_{1}z)+b_{6}\exp(-m_{2}z)\\ \;\;\;\;\;\;\;\;\;\;\;\;\;\;\;\;\;\;\;\;\;\;\;\;\;\;\;\;\;\;\;\;\;\;\;\;\;\;\;\;\;\;\;\;\;\;\;\;\;\;\;\;\;\;\;\;\;\;\;\;\;\;\;\;\;\;\;\;\;\;\;\;\;+b_{7}\exp(-m_{3}z)+b_{8}\exp(-m_{4}z)) \end{array}$$

(47) $$\phi =\exp(-m_{1}z)+\varepsilon e^{iwt}(b_{1}\exp(-m_{1}z)+b_{2}\exp(-m_{2}z))$$

For this particular boundary layer flow, the skin friction co-efficient, the Nusselt number, and the Sherwood number are all crucially significant physical characteristics. The following is a definition and determination of each of these parameters:

### 3.1. Skin Friction

Very important physical parameter at the boundary is the skin friction which is given in the non-dimensional form and derives as

(48) $$\tau ={\left(\frac{\partial q}{\partial z}\right)}_{z=0}=-\left(\left(b_{9}m_{1}+b_{10}m_{3}+b_{11}m_{5}\right)+\;\varepsilon e^{iwt}\left(\begin{array}{l} b_{12}m_{1}+b_{13}m_{2}+\\ b_{14}m_{3}+b_{15}m_{4}+\;\;b_{16}m_{5}+b_{17}m_{6} \end{array}\right)\right)$$

### 3.2. Nusselt Number

Another physical parameter like rate of heat transfer, in the form of Nusselt number, is expressed by

(49) $$Nu=-{\left(\frac{\partial \theta }{\partial z}\right)}_{z=0}=\left(\left(b_{3}m_{1}+b_{4}m_{3}\right)+\varepsilon e^{iwt}(b_{5}m_{1}+b_{6}m_{2}+b_{7}m_{3}+b_{8}m_{4})\right)$$

### 3.3. Sherwood Number

The rate of mass transfer in the form of Sherwood number is also derived by

(50) $$Sh=-{\left(\frac{\partial \Phi }{\partial z}\right)}_{z=0}=m_{1}+\varepsilon e^{iwt}(b_{1}m_{1}+b_{2}m_{2}\;)$$

## 4. Results and Discussion

**Figure 2:**
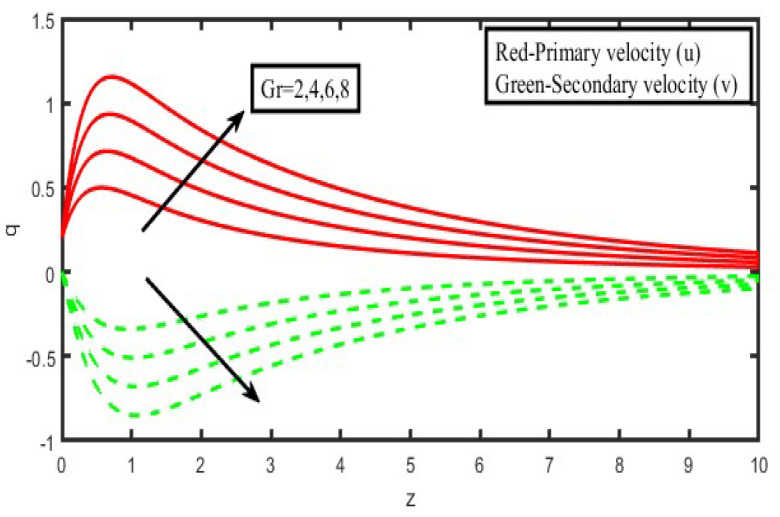
Velocity profiles for u and v for thermal Grashof number (Gr).

**Figure 3:**
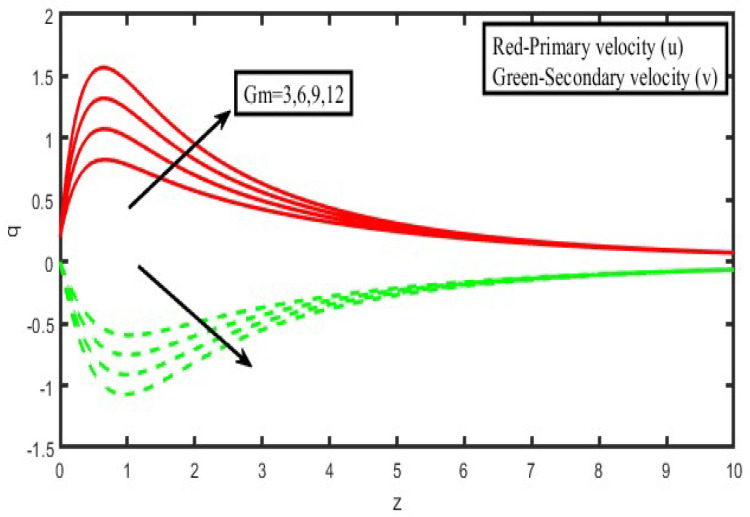
Velocity profiles for u and v for modified Grashof number (Gm).

**Figure 4:**
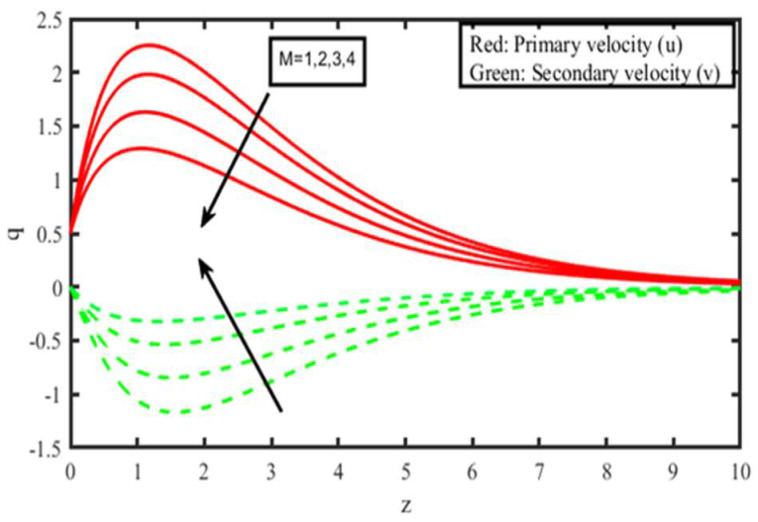
Velocity profiles for u and v for Magnetic field parameter (M).

**Figure 5:**
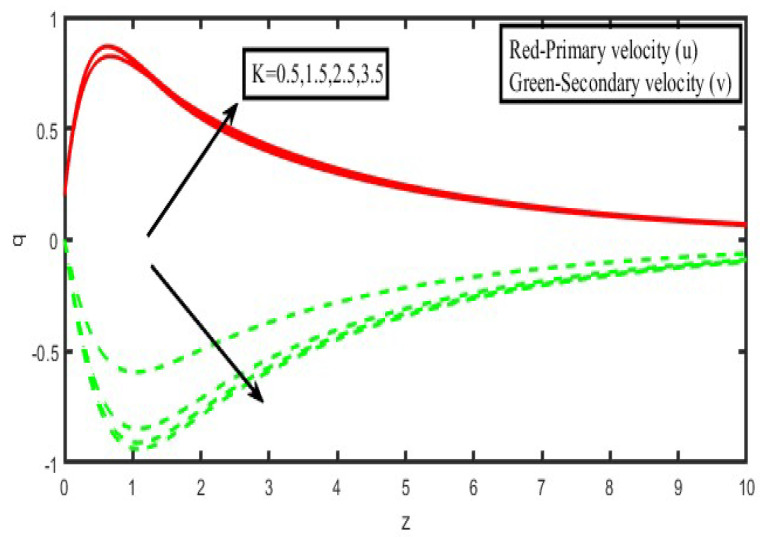
Velocity profiles for u and v for Permeability of porous media (K).

**Figure 6:**
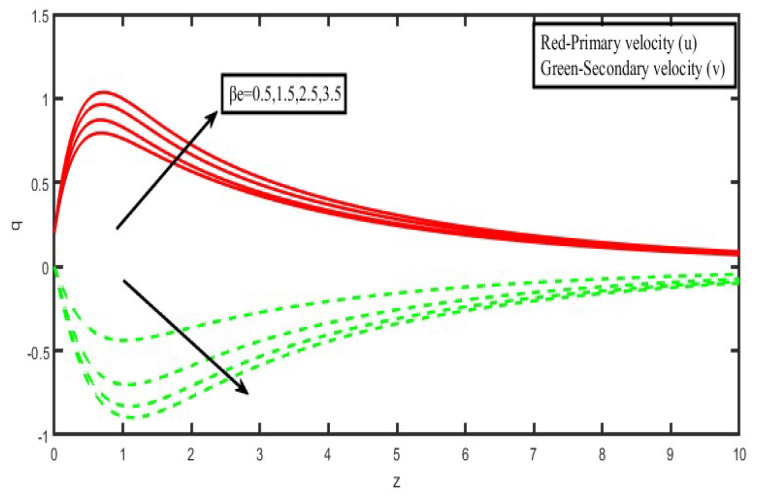
Velocity profiles for u and v for Hall parameter (β_e_).

**Figure 7:**
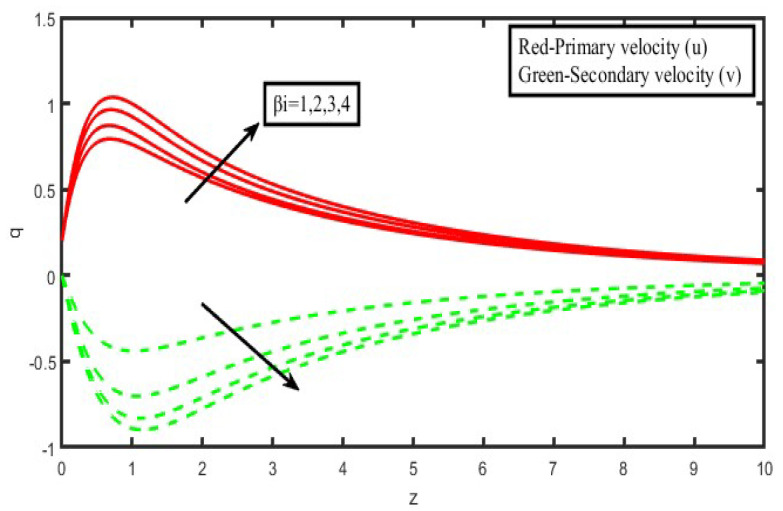
Velocity profiles for u and v for Hall parameter (β_i_).

**Figure 8:**
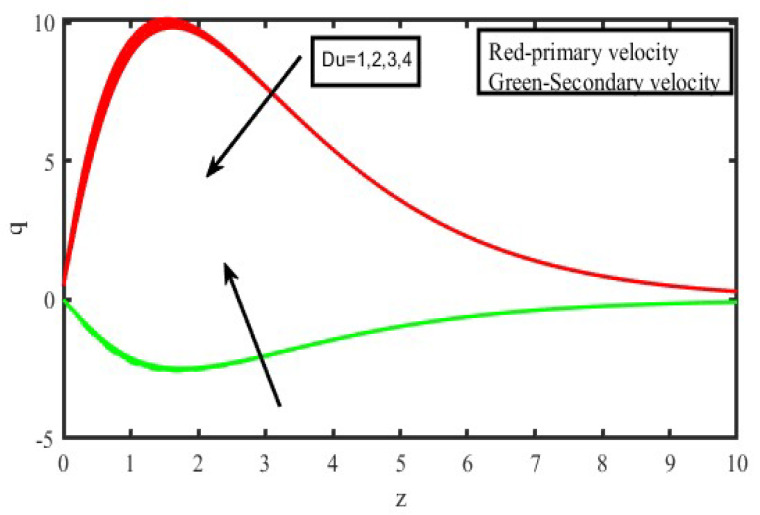
Velocity profiles for u and v for Diffusion thermo specification (D_u_).

**Figure 9:**
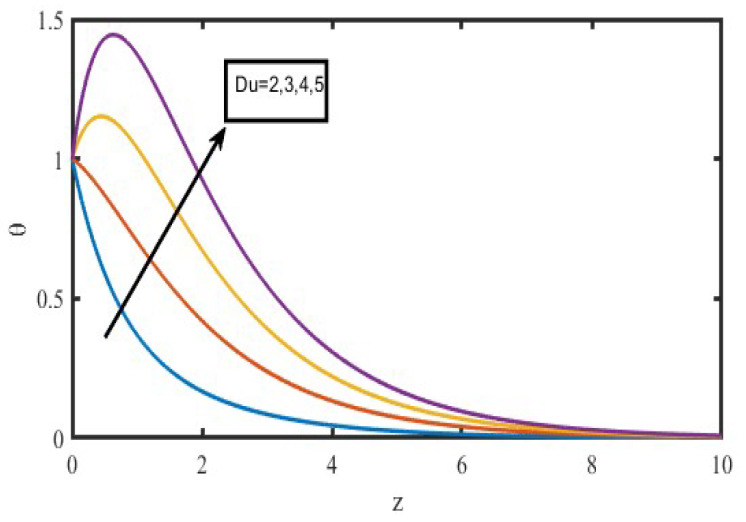
Temperature profiles for Diffusion thermo specification (D_u_).

**Figure 10:**
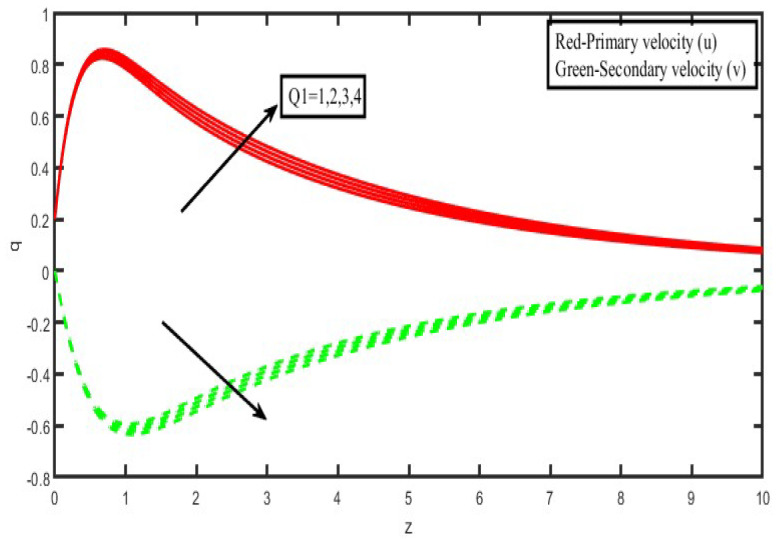
Velocity profiles for u and v for Radiation Absorption (Q_1_).

**Figure 11:**
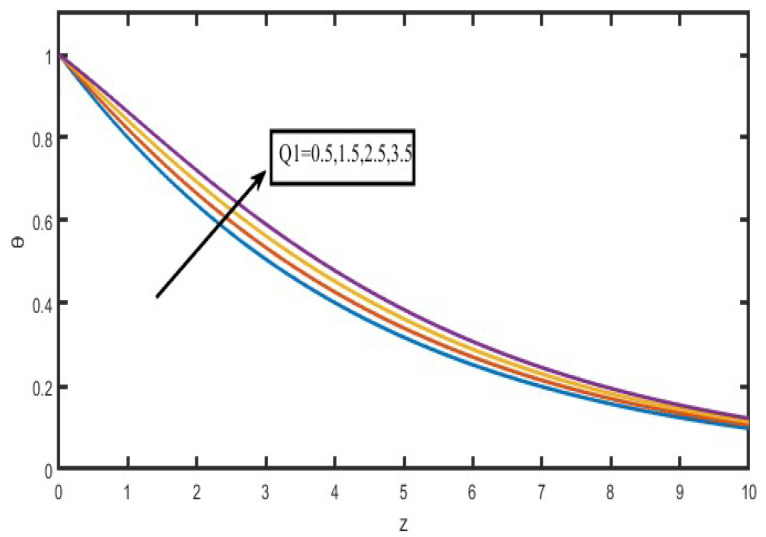
Temperature profiles for Radiation absorption (Q_1_).

**Figure 12:**
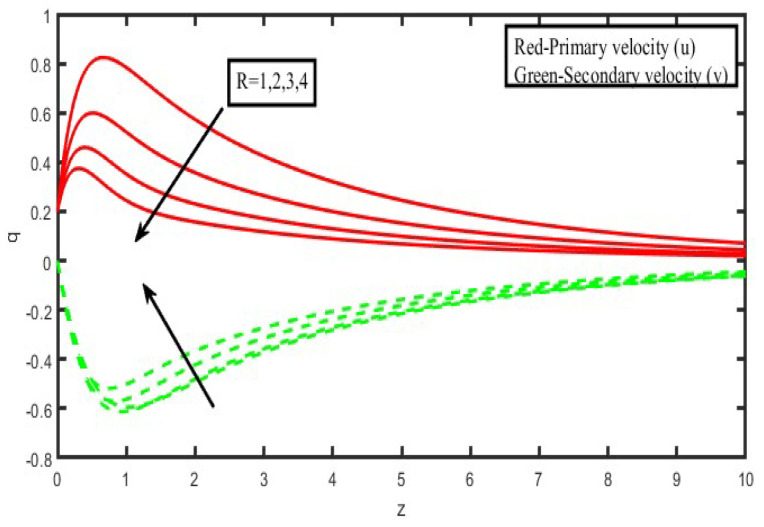
Velocity profiles for u and v for Rotation specification (R).

**Figure 13:**
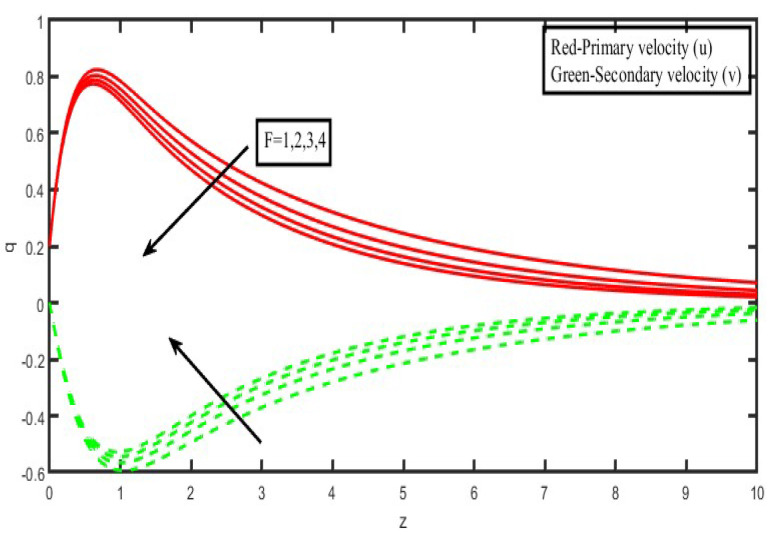
Velocity profiles for u and v for Radiation parameter (F).

**Figure 14:**
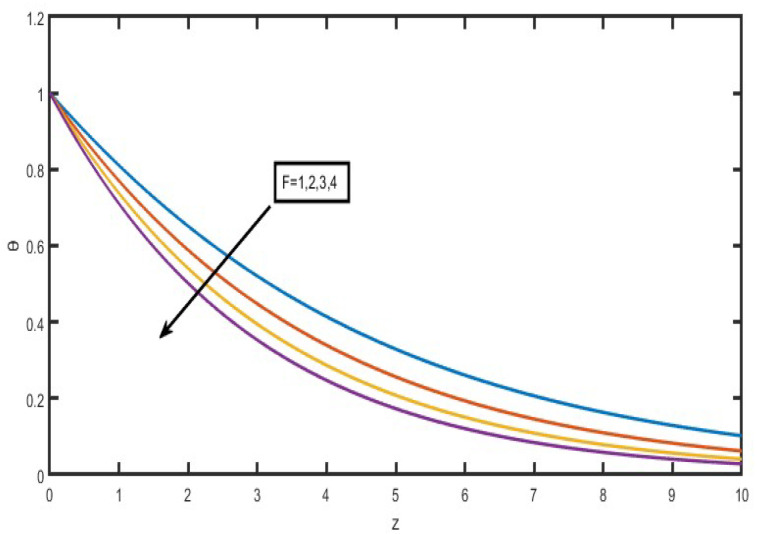
Temperature profiles for Radiation parameter (F).

**Figure 15:**
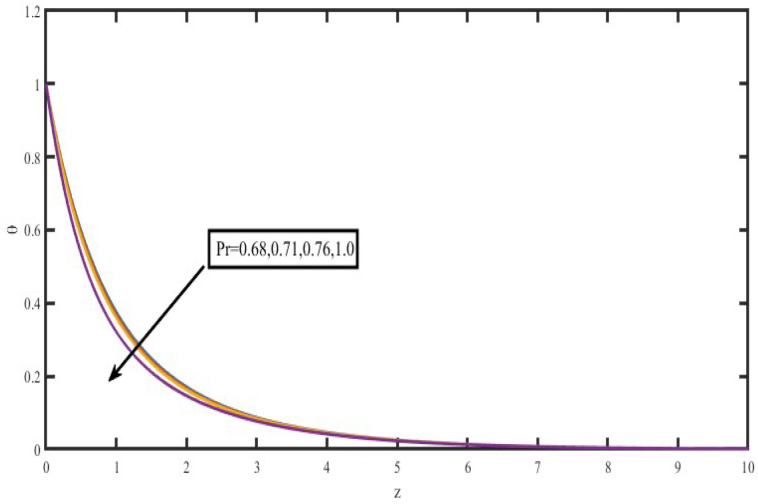
Temperature profiles for Prandtl number (Pr).

**Figure 16:**
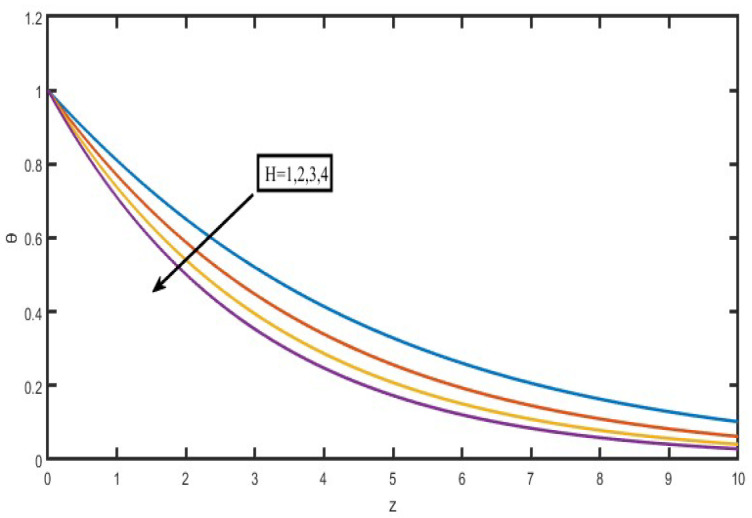
Temperature profiles for Heat absorption Parameter (H).

**Figure 17:**
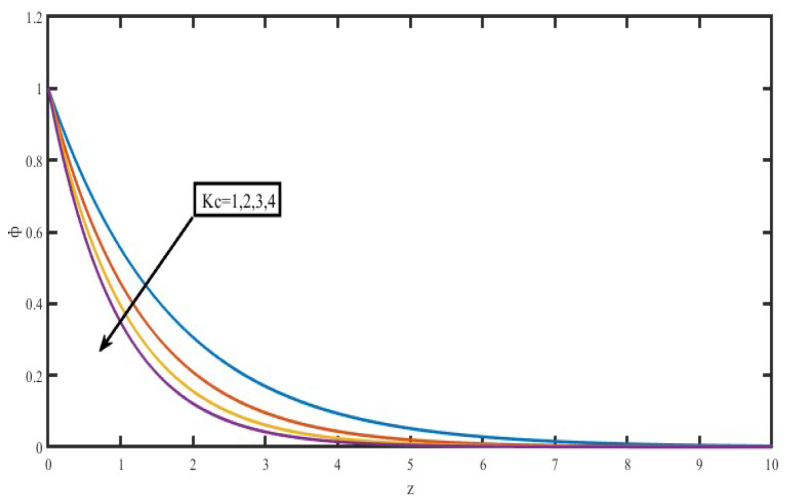
Concentration profiles for Chemical reaction (K_c_).

**Figure 18:**
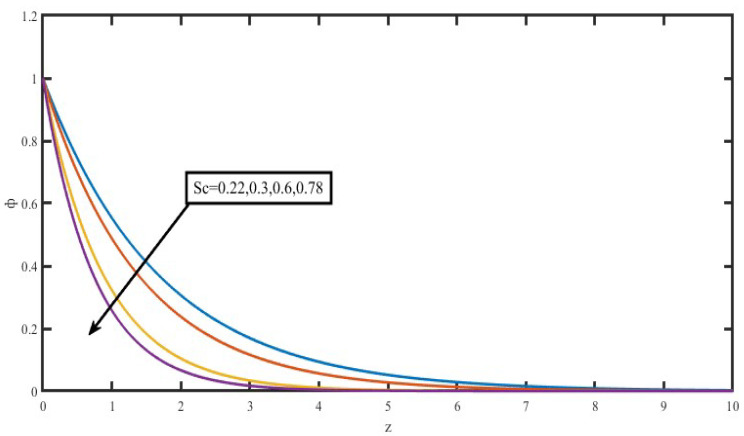
Concentration profiles for Schmidt number (S_c_).

The present investigation aims to investigate the effects of radiation absorption, Hall, and ion slip on the uncertain free convective flow of an electrically conducting fluid that is viscous and incompressible over an unbounded vertical porous plate. At the same time, a uniform transverse magnetic field is present. A regular perturbation approach is used to find solutions to the governing equations of the flow field when the Eckert number Ec is small. The closed-form solutions for the velocity, temperature, and concentration have been derived analytically. Its behaviour is computationally addressed concerning various flow characteristics such as the Hartmann number (M), the Hall parameter (e), the ion slip parameter (e), the thermal Grashof number (Gr), the mass Grashof number (Gm), the permeability of porous media (K), the radiation absorption criterion (Q_1_), the diffusion thermo criterion (Du), the Prandtl number (Pr). For computational intention, we are setting up the values A = 2, ↋ = 0:001; U_0_ = 0.1, while the parameters being M = 2, K = 0.5, R = 1, S = 0.5, Gr = 5, Gm = 3, be = 1, bi = 0.2, Pr = 0.71, H = 1, Sc = 0.22, Kc = 1, Du = 2, Q_1_ = 0.5, t = 2 fixed over the range. Figure 2, Figure 3, Figure 4, Figure 5, Figure 6, Figure 7, Figure 8, Figure 9, Figure 10, Figure 11, Figure 12, Figure 13, Figure 14, Figure 15, Figure 16, Figure 17 and Figure 18 are shown here, with their respective velocities, temperatures, and concentrations represented as distributions. The stresses, Nusselt number, and Sherwood number at the plate are analyzed numerically, explained with governing factors, and summarized in the Table 1, Table 2 and Table 3. The outcomes of this investigation, as indicated in Table 4, are consistent with the findings of the prior study [10], which Raghunath and his colleagues conducted.

Figure 2 and Figure 3 illustrate the consequences of the thermal buoyancy force, denoted by Gr, and the concentration buoyancy force, characterized by Gm. Increasing the thermal and mass Grashof numbers causes fluid velocity in the principal flow direction inside the boundary layer area. This is the case regardless of whether the Grashof number is increased. This is because an increase in both the thermal and the solutal Grashof number causes a rise in buoyancy effects, and these effects cause more flow in the direction that is already the dominant flow. There is a reverse flow occurring in the direction of the secondary flow. When the temperature and concentration of Grashof numbers are increased, the secondary velocity profiles rise in the boundary layer area further away from the plate. At the same time, they fall closer to the plate. It should also be observed that there is no reversal flow in the secondary flow direction when neither heat nor concentration buoyancy forces are present. This is something that should be taken into consideration. This indicates that the forces associated with buoyancy and the movements of the free stream are to blame for the induction of reverse flow.

We see a decrease in the size of the velocity components u and v, as well as a reduction of the velocity of resultant velocity when the strength of the magnetic field is increased, as shown in Figure 4. Because the effects of a transverse magnetic field on an electrically conducting fluid generate a piezoresistive force (also known as the Lorentz force), which is analogous to the drag force, growing M causes the drag force to raise, which in turn causes the motion of the fluid to slow down as a direct result of the increased drag force. As the passage of time causes the permeability factor (K) to grow, Figure 5 demonstrates that the subsequent velocity component u becomes more concentrated while increasing in height. When K is made higher, the consequent velocity is also pushed higher, increasing the thickness of the momentum boundary layer. A decrease in porosity leads to an increase in fluid speed that is less visible once measured within the flow zone filled by the liquid.

Both Figure 6 and Figure 7 illustrate how the Hall current parameters, denoted by (βe), and the ion-slip parameter, denoted by (βi), affect the fluid velocity. It is clear from looking at Figure 4 and Figure 5 that as the Hall and ion-slip parameters are increased, the fluid velocity in the primary flow direction decreases in the boundary layer region that is close to the plate, but it increases in the boundary layer region that is further away from the plate. This phenomenon can be seen in both of these figures. The fluid velocity in the secondary flow direction increases everywhere in the boundary layer area due to an increase in the hall parameter, except for a narrow region where it disappears. This is because the Hall current is generated as a result of the spiralling of conducting fluid particles around magnetic lines of force, which have the potential to create secondary motion in the flow field. The nature of the ion-slip current on the fluid flow in the secondary flow direction is shown to be the exact opposite of that of the Hall current.

The influence of the diffusion thermo specification can be seen in Figure 8 and Figure 9, which depict the velocity and temperature profiles, respectively. Figure 8 demonstrates the effect of the Dufour number on the primary and secondary velocities, which may be found on this page. It shows that greater values of the Dufour number cause the initial momentum to increase, but it indicates that the behaviour of the second velocity is the exact reverse of what one would expect. This pattern is a direct result of the generation of energy flow, which makes it possible for the rate of motion to pick up speed. Figure 9 makes it clear that an elevation in the values of the Dufour number leads to an accompanying rise in the temperature of the fluid. This is shown by the fact that an increase in the importance of the Dufour number can be seen. The generation of energy flow, which causes a temperature rise, is responsible for this occurrence.

The impact of the radiating absorption characteristic is seen in Figure 10 in both the primary and secondary velocity directions. According to the statistics, the main velocity graphs increase when there is an increase in radiating absorption. Still, the secondary velocity graphs go down when there is a drop in radiating absorptions over the whole liquid region. It was interesting to see how an increase in the radiating absorption characteristics might result in a rise in velocity. Inside the border layers, a description of the effects of the radiation-absorption parameter on the temperatures is depicted. As seen in Figure 11, it has become abundantly clear that the temperature distributions are rising functions of the absorbed radiation. In contrast, the inverse pattern was found when the rotation parameter was used, as shown in Figure 12.

The influence of the radiation factor on the velocity and temperature is shown in Figure 13 and Figure 14. It is clear from looking at Figure 13 shows that a rise in the radiation parameter causes a decrease in the essential velocity, but it has the opposite effect on the secondary velocity. As a consequence of this, the resulting velocity decreases as the value of R increases over the whole of the area that is occupied by the fluid. The influence of the thermal radiation parameter is quite essential when it comes to temperature profiles. It has been discovered that there is a negative correlation between the increase in the radiation parameter and the temperature (Figure 14). As a result of the action of the radiation parameter, it is also possible to observe that the thickness of the thermal boundary layer quickly decreases.

Figure 15 illustrates how the Prandtl number (Pr) influences temperature profiles when certain fluids are present. These fluids include hydrogen (Pr = 0.684), air (Pr = 0.71), carbon dioxide (Pr = 0.72), and water (Pr = 1.0). According to the data in this figure, a rise in the Prandtl number results in a drop in temperature over the whole flow field. This agrees with the theory that the thickness of the thermal boundary layer decreases as the Prandtl number increases. Figure 16 displayed the differences in temperature profiles that resulted from using various values for the heat source specification H. Bringing down the temperature of the flow field, increasing the value of the heat source parameter. This may occur because of the fluid’s elastic quality.

Figure 17 and Figure 18 illustrate the chemical reaction parameter Kc’s influence and the effect that the Schmidt number has on the concentration distribution. Figure 17 demonstrates an increase in the value of the parameter for the chemical reaction. The concentration profiles were quickly decreased due to Kc. As the number of chemically responsive factors increases, the number of chemical reactions that influence the quantities of solutal particles also increases, which causes the concentration domain to decrease. The end effect of the chemical reaction is a reduction in the breadth of the solute border stratum. In Figure 18, the concentration gradient is shown as having decreased over the whole flow field as the Schmidt number Sc increased. This demonstrates that heavier diffusing species have a higher impact on inhibiting the concentration dispersion of the flow field.

The values for the magnitudes of skin friction are summarized in Table 1, which may be found below. An increase in the Hartmann number will be introduced to decrease the amount of skin friction. This application uses second-grade fluid because of its elastic nature, which reduces the frictional drag caused by the liquid. It is explored what happens to similar behavior when the rotation factor, the Prandtl number, the heat source factor, the Schmidt number, the chemical reaction factor, and the amount of time are all enhanced. An increase in the permeability parameter or the second-grade fluid parameter will further improve the expansion of skin friction in intensity on the edge of the surface. The analogous activity is explored for the same when an increase in the thermal Grashof number, mass Grashof number, Hall and ion aspects that affect them are enhanced on the boundary of the surface.

As a consequence, a rise in the Nusselt number also results from an increase in the radiation parameter, the Prandtl number, and the heat source parameters, as shown in Table 2. The radiation absorption parameter and the frequency oscillations of the radiation need to be increased for it to be diminished. According to Table 3, an increase in the Schmidt number, the chemical reaction parameter, the frequency of fluctuations, or non-dimensional time all contribute to strengthening the Sherwood number as time proceeds. In Table 4 expressed as the results of the present study are pretty congruent with the findings of the previous study by Raghunath and colleagues [10]. Recently, investigations regarding nanomaterials are listed in Refs. [33,34].

**Table 1:** Skin friction.

Gr	Gm	βe	βi	Pr	H	Sc	Kc	R	Du	M	K	S	Q_1_	τ
5	3	1	0.2	0.71	1	0.22	1	1	1	2	0.5	0.5	0.5	2.1521
5	3	1	0.2	0.71	1	0.22	1	1	1	3	0.5	0.5	1	2.7852
5	3	1	0.2	0.71	1	0.22	1	1	1	2	1.0	05	1	1.4521
5	3	1	0.2	0.71	1	0.22	1	1	1	2	1.5	0.5	1	3.7852
5	3	1	0.2	0.71	1	0.22	1	1	1	2	0.5	0.5	2	3.5211
5	3	1	0.2	0.71	1	0.22	1	1	1	2	0.5	1.0	3	2.0332
5	3	1	0.2	0.71	1	0.22	1	1	1	2	0.5	1.5	1	2.7852
9	3	1	0.2	0.71	1	0.22	1	1	1	2	0.5	0.5	1	3.7852
12	3	1	0.2	0.71	1	0.22	1	1	1	2	0.5	0.5	1	2.7852
5	6	1	0.2	0.71	1	0.22	1	1	1	2	0.5	0.5	1	5.4621
5	9	1	0.2	0.71	1	0.22	1	1	1	2	0.5	0.5	1	3.3221
5	3	2	0.2	0.71	1	0.22	1	1	1	2	0.5	05	1	3.0214
5	3	3	0.2	0.71	1	0.22	1	1	1	2	0.5	0.5	1	4.7852
5	3	1	0.4	0.71	1	0.22	1	1	1	2	0.5	0.5	1	2.0324
5	3	1	0.6	0.71	1	0.22	1	1	1	2	0.5	0.5	1	3.7852
5	3	1	0.2	5.0	1	0.22	1	1	1	2	0.5	0.5	1	3.0125
5	3	1	0.2	7.0	1	0.22	1	1	1	2	0.5	05	1	2.7852
5	3	1	0.2	0.71	2	0.22	1	1	1	2	0.5	0.5	1	1.3214
5	3	1	0.2	0.71	3	0.22	1	1	1	2	0.5	0.5	1	1.7852
5	3	1	0.2	0.71	1	0.25	1	1	1	2	0.5	0.5	1	2.7852
5	3	1	0.2	0.71	1	0.30	1	1	1	2	0.5	0.5	1	2.1255
5	3	1	0.2	0.71	1	0.22	2	1	1	2	0.5	05	1	2.7852
5	3	1	0.2	0.71	1	0.22	4	1	1	2	0.5	0.5	1	2.1254
5	3	1	0.2	0.71	1	0.22	1	2	1	2	0.5	0.5	1	2.7852
5	3	1	0.2	0.71	1	0.22	1	2	2	2	0.5	0.5	1	2.7852
5	3	1	0.2	0.71	1	0.22	1	2	3	2	0.5	0.5	1	2.6221

**Table 2:** Nusselt number.

F	Q_1_	H	Pr	ω	Du	Nu
2	0.5	1	0.71	π/6	1	1.1251
3	0.5	1	0.71	π/6	1	1.7852
1	1	1	0.71	π/6	1	−0.12541
1	2	1	0.71	π/6	1	−1.65514
1	0.5	2	0.71	π/6	1	1.4521
1	0.5	3	0.71	π/4	1	1.4021
1	0.5	1	5.0	π/3	1	1.2785
1	0.5	1	7.0	π/6	1	1.3210
1	0.5	1	0.71	π/4	1	1.4521
1	0.5	1	0.71	π/3	1	1.0321
1	0.5	1	0.71	π/3	2	1.7520
1	0.5	1	0.71	π/3	3	1.0321

**Table 3:** Sherwood number.

Sc	Kc	ω	Sh
0.3	1	π/6	0.6521
0.6	1	π/6	0.9852
0.22	2	π/6	0.1201
0.22	3	π/6	0.5200
0.22	1	π/4	0.4520
0.22	1	π/3	1.4520

**Table 4:** Comparison of results for primary velocity (A = 5, *n* = 0.5, t = 0.5, є = 0.01, U_0_ = 0.5, Sc = 0.22, Kc = H = Q_1_ = F = 1, Du = 0).

M	K	Gr	Gm	Previous Results Raghunath et al. [10]	Present Values
2	0.5	5	3	0.703484	0.752100
3				0.452455	0.452114
4				0.302545	0.302144
	1.0			0.797822	0.785210
	1.5			0.835478	0.842011
		8		0.934587	0.962214
		12		1.161458	1.122348
			5	0.780458	0.788752
			7	0.851458	0.852147

## 5. Conclusions

i. By increasing the magnetic field’s strength, the rotating speed, or the properties of the second-grade fluid, the velocity produced may be reduced.
ii. Permeability of the porous medium may be enhanced to have the same effect as increases.
iii. The velocity is intended to decrease if the pore size of a porous medium decreases, which is the same behaviour seen in the case of radiation.
iv. An increase in the Hall parameter and the ion slip parameter over the fluid region contribute to the rise in the ultimately produced velocity.
v. The resultant velocity is affected by the thermal and solute buoyancy forces, and it will continue to increase in intensity until it hits a significant threshold.
vi. As the diffusion thermo and radiation absorption parameter, the resulting velocity and temperature.
vii. When both the heat source parameter and radiation increase, the temperature distribution becomes more concentrated. The fluid medium has decreased concentration due to the chemical reaction and the Schmidt number.

## Data Availability

All the data are clearly available in the manuscript.

## Nomenclature

x, y	dimensional co-ordinates (m)
u, v	velocity components along x, y directions (m/s)
A	real positive constant
B_0_	applied magnetic field (A/m)
C	non- dimensional fluid concentration (kg/m^3^)
C_p_	specific heat at a constant pressure (J/kg. K)
C_w_	the uniform concentration of the fluid at the plate (kg m^−3^)
C_∞_	the concentration of the fluid far away from the plate (kg m^−3^)
D	coefficient of mass diffusivity (m^2^/s)
D_M_	Chemical molecular diffusivity; m^−2^·s^−1^
g	acceleration due to gravity (m s^−2^)
Gr	thermal Grashof number
Gm	mass Grashof number
B	magnetic field vector (A/m)
E	electric field vector (c)
V	velocity vector (m/s)
Q_0_	heat source parameter
J	current density vector (A/m^2^)
J_x_, J_y_	current densities along x and y directions
k	permeability of porous medium (m^2^)
k_1_	thermal conductivity (W/m K)
K	permeability parameter
Kc	chemical reaction parameter (w/mk)
K_l_	chemical reaction rate constant
m	Hall parameter
M	Hartmann number
N	constant
Nu	local Nusselt number
P_e_	electron pressure (Pascal)
Pr	Prandtl number
q_m_	local surface mass flux (kg s^−1^ m^−2^)
q_w_	local surface heat flux (W m^−2^)
R	rotation parameter
S	second grade fluid
Sc	Schmidt number
Sh	local Sherwood number
Q_1_	radiation absorption parameter
t	time (s)
T_w_	the uniform temperature of the fluid at the plate (K)
T_∞_	the temperature of the fluid far away from the plate (K)
u_0_	plate velocity (m s^−1^)
w	slip velocity (m s^−1^)
w_0_	scale of suction velocity
q_r_	radiative heat flux
**Greek symbols**	
β	coefficient of thermal expansion of the fluid
β*	coefficient of mass expansion of the solid
θ	non-dimensional temperature (K)
Φ	non-dimensional concentration (mol/m^3^)
ν	kinematic viscosity (m^2^/s)
ρ	fluid density (Kg/m^3^)
σ	electrical conductivity (S/m)
Ω	angular velocity (s^−1^)
τ_w_	local wall shear stress (pascal)
τ	local skin friction coefficient
τ_e_	electron collision time (s)
ω_e_	cyclotron frequency (e/mB)
**Subscripts and superscripts**	
e	electrons
i	ions
w	conditions on the wall
∞	free stream conditions
